# Supplementary material for: Comprehensive Analysis of 1-Year-Old Female Apolipoprotein E-Deficient Mice Reveals Advanced Atherosclerosis with Vulnerable Plaque Characteristics
Source: Int J Mol Sci. 2024 Jan 22;25(2):1355. doi: 10.3390/ijms25021355 (PMC10816800; doi:10.3390/ijms25021355)
Supplement: Supplementary file 1 [file ijms-25-01355-s001.zip › ijms-2824307-supplementary.pdf]

Supplementary Material

Comprehensive analysis of 1-year-old apolipoprotein E-deficient mice reveals advanced atherosclerosis with vulnerable plaque characteristics.

Sotirios Kotsovilis[1,†], Maria Salagianni[1,†], Aimilia Varela[2], Constantinos H. Davos[2], Ioanna E. Galani[1], Evangelos Andreakos[1*]

[1]Laboratory of Immunobiology, Center for Clinical, Experimental Surgery, and Translational Research, Biomedical Research Foundation, Academy of Athens, Athens, Greece

[2]Cardiovascular Research Laboratory, Clinical, Experimental Surgery, and Translational Research Center, Biomedical Research Foundation, Academy of Athens, Athens, Greece

*** Correspondence:** Evangelos Andreakos: vandreakos@bioacademy.gr

Supplementary Figures

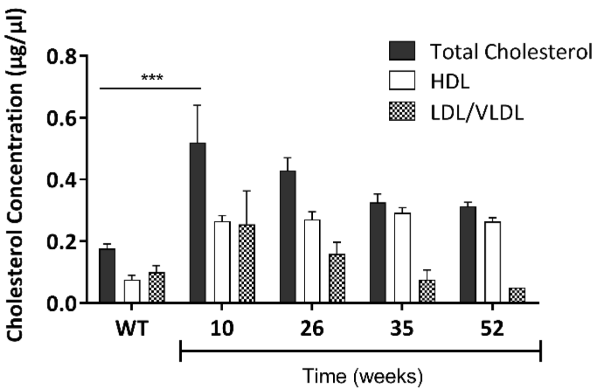

Supplementary Figure 1. Serum cholesterol lipid profiles. Serum concentrations of total cholesterol (black), HDL (white), LDL/VLDL (dotted) in 10-, 26- and 52-week-old *Apoe*$^{-/-}$ mice fed on normal chow diet (*n* = 10, 9, 9 and 3 mice for each group, respectively). 10-week old wild-type (WT) mice (n=3) fed a normal chow diet were used as controls. Statistical comparisons between groups were performed using the 2-wayAnova. *** $p \leq 0.001$. Whenever *p* value is not mentioned, differences between groups were not statistically significant ($p > 0.05$).

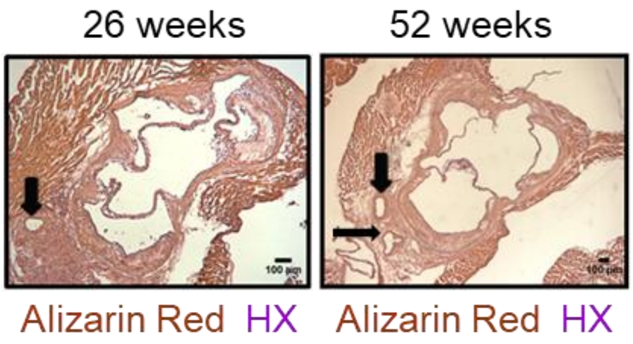

Supplementary Figure2. Development of calcification in coronary arteries. Representative light photomicrographs of Alizarin Red S stained serial cross sections from the aortic root of 26- and 52-week-old mice. No calcification was detected in coronary arteries (black arrows) at 26- and 52-week-old mice after Alizarin Red S staining.

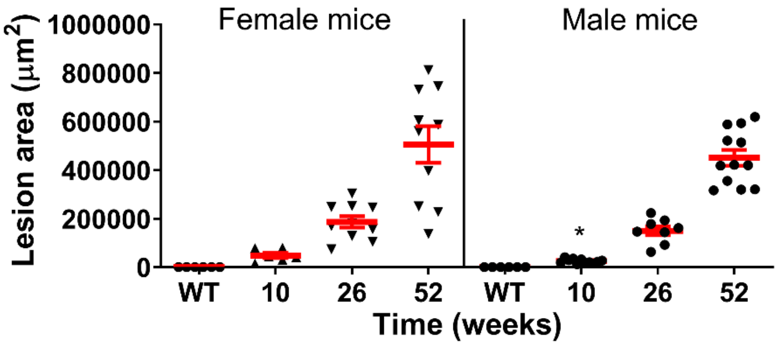

Supplementary Figure 3. Morphometric analysis of atherosclerotic lesion area of male mice compared to female mice. Atherosclerotic lesion area of male mice and comparison with female mice determined using morphometric analysis of Oil Red O-stained serial cross sections from the aortic root of wild-type C57BL/6 mice (WT), and 10-, 26- and 52-week-old *Apoe*$^{-/-}$ mice (n = 6, 6, 10, 10, 6, 11, 8, 12 mice for each group, respectively). Mean value ± standard error of the mean (SEM) are displayed in red. Statistical comparisons between same age groups were performed using

the Mann-Whitney *U* test. * $p \leq 0.05$. Whenever *p* value is not mentioned, differences between groups were not statistically significant ($p > 0.05$).

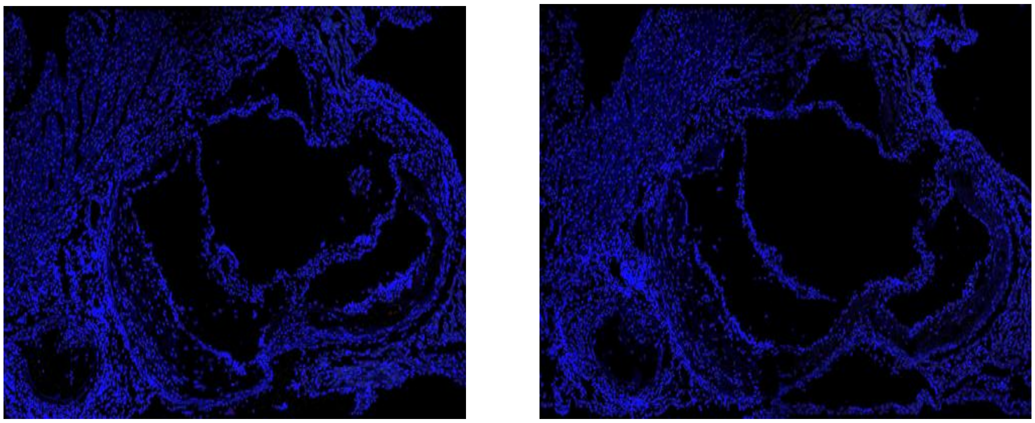

a b

Supplementary Figure 4.**Isotype and unstained control.**(**a**) Absence of positive signal in an isotype control section incubated with rat IgG in the aortic root of a female *Apoe*$^{-/-}$mouse aged 52 weeks.(b) Absence of positive signal in an unstained control section in the aortic root of a female *Apoe*$^{-/-}$aged 52 weeks.
